# Magnetic Resonance Imaging and Spectroscopy Methods to Study Hepatic Glucose Metabolism and Their Applications in the Healthy and Diabetic Liver

**DOI:** 10.3390/metabo12121223

**Published:** 2022-12-05

**Authors:** Ayhan Gursan, Jeanine J. Prompers

**Affiliations:** Center for Image Sciences, University Medical Center Utrecht, 3584 CX Utrecht, The Netherlands

**Keywords:** glucose homeostasis, liver metabolism, diabetes, magnetic resonance spectroscopy, hyperpolarization

## Abstract

The liver plays an important role in whole-body glucose homeostasis by taking up glucose from and releasing glucose into the blood circulation. In the postprandial state, excess glucose in the blood circulation is stored in hepatocytes as glycogen. In the postabsorptive state, the liver produces glucose by breaking down glycogen and from noncarbohydrate precursors such as lactate. In metabolic diseases such as diabetes, these processes are dysregulated, resulting in abnormal blood glucose levels. Magnetic resonance imaging (MRI) and magnetic resonance spectroscopy (MRS) are noninvasive techniques that give unique insight into different aspects of glucose metabolism, such as glycogenesis, glycogenolysis, and gluconeogenesis, in the liver in vivo. Using these techniques, liver glucose metabolism has been studied in regard to a variety of interventions, such as fasting, meal intake, and exercise. Moreover, deviations from normal hepatic glucose metabolism have been investigated in both patients with type 1 and 2 diabetes, as well as the effects of antidiabetic medications. This review provides an overview of current MR techniques to measure hepatic glucose metabolism and the insights obtained by the application of these techniques in the healthy and diabetic liver.

## 1. Introduction

### 1.1. Hepatic Glucose Metabolism

**Figure 1:**
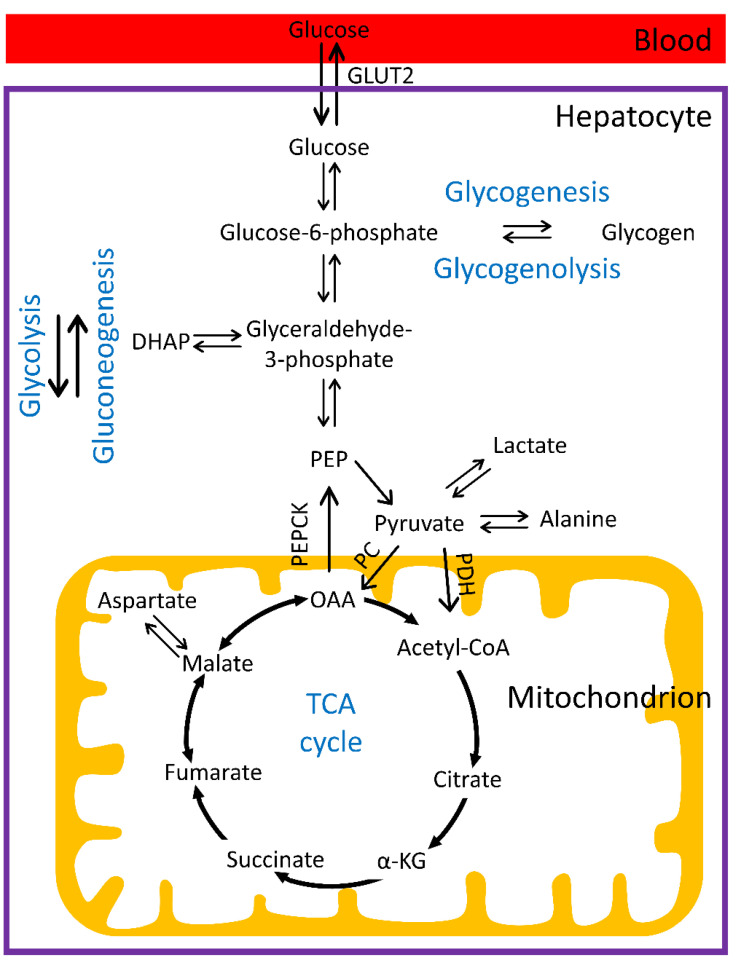
Schematic of hepatic glucose metabolism. α-KG, alpha-ketoglutarate; DHAP, dihydroxy acetone; GLUT2, glucose transporter 2; OAA, oxaloacetate; PC, pyruvate carboxylase; PDH pyruvate dehydrogenase; PEP, phosphoenolpyruvate; PEPCK, phosphoenolpyruvate carboxykinase; TCA, tricarboxylic acid.

The liver plays a major role in the maintenance of glucose homeostasis in the body. In the postprandial state, hepatic glucose uptake accounts for ~25% of whole-body glucose uptake [1,2]. The principal transporter that facilitates glucose movement between the blood and hepatocytes is glucose transporter 2 (GLUT2) [3]. Inside hepatocytes, glucose is phosphorylated to glucose-6-phosphate (G6P) by glucokinase (Figure 1). Due to the negative charges on the phosphoryl group, G6P is not a substrate for glucose transporters and does not cross the membrane. G6P cannot be stored directly, as high concentrations would disrupt the osmotic balance in the cell and cause damage. Therefore, G6P is converted to glycogen, which is a non-osmotically active polymer, in a process termed glycogenesis (Figure 1). The majority of glucose taken up in the liver is stored as glycogen through glycogenesis [4]. Alternatively, G6P can enter the glycolytic pathway to produce pyruvate. After transport into the mitochondria, pyruvate can then be converted to acetyl-CoA by pyruvate dehydrogenase (PDH) and further metabolized oxidatively in the tricarboxylic acid (TCA) cycle (Figure 1). However, the liver is thought to have very low rates of PDH flux and non-PDH sources of acetyl-CoA, such as from fatty acid oxidation, dominate oxidative metabolism in the liver [5]. The major cytosolic fate of pyruvate produced by glycolysis is the reduction to lactate or transamination into alanine (Figure 1). Alternatively, pyruvate can also be used as substrate for de novo lipogenesis, but this pathway is not covered in this review.

In the postabsorptive phase, when blood glucose levels are low, hepatic glycogen is broken down to glucose (Figure 1), a process termed glycogenolysis, which triggers glucose release into the blood circulation. In addition to glycogenolysis, the liver can also synthesize glucose from noncarbohydrate precursors, such as pyruvate, lactate, alanine, and glycerol, a process called gluconeogenesis. Pyruvate is carboxylated to oxaloacetate by pyruvate carboxylase (PC), and phosphoenolpyruvate carboxykinase (PEPCK) then converts oxaloacetate to phosphoenolpyruvate (PEP), which is a critical substrate of gluconeogenesis (Figure 1). Both lactate and alanine, produced from pyruvate as described above, are first converted back into pyruvate to enter the gluconeogenic pathway. Skeletal muscle is a major source of plasma lactate and alanine. Lactate and alanine produced and exported by the muscles is taken up by the liver to regenerate glucose through gluconeogenesis. Glycerol is mainly derived from fat metabolism and can be converted to dihydroxyacetone phosphate (DHAP), which is an intermediate in the gluconeogenic pathway (Figure 1).

### 1.2. Regulation of Glucose Homeostasis and Distortions in Diabetes

The hormone insulin is secreted by the pancreas when blood glucose levels increase above normal values, i.e., hyperglycemia, to promote glucose uptake in the liver and skeletal muscle. In hypoglycemia, the hormone glucagon is released from the pancreas and raises circulating glucose levels primarily via hepatic glycogenolysis, and to a lesser degree by gluconeogenesis in the liver. However, in type 1 and type 2 diabetes, these processes are dysregulated, resulting in hyperglycemia or hypoglycemia, where the latter is usually caused by antidiabetic medication. In type 1 diabetes (T1D), also known as insulin-dependent diabetes, the pancreas produces little or no insulin, because of autoimmune destruction of insulin-producing β-cells. As there is still no cure for T1D, current treatments involve exogenous insulin administration (insulin therapy), as well as diet and lifestyle changes. Normal blood sugar levels can be achieved with insulin therapy, but the dosage must be adjusted for calorie consumption and physical activity. In T1D patients, hypoglycemia may not trigger a typical glucagon response, and exogenous glucagon can be needed to treat severe hypoglycemia. In type 2 diabetes (T2D), both insufficient insulin secretion in the pancreas and an impaired insulin sensitivity of the liver and skeletal muscle contribute to the observed hyperglycemia. Newly diagnosed T2D patients are usually treated with oral antidiabetic medication in combination with diet and lifestyle changes. Improved diet and lifestyle can potentially reverse T2D, but when the disease progresses, T2D patients may also become dependent on insulin therapy.

### 1.3. Magnetic Resonance Imaging and Spectroscopy

Magnetic resonance imaging (MRI) and magnetic resonance spectroscopy (MRS) are noninvasive techniques that hold great promise for the assessment of different aspects of liver metabolism, such as glycogenesis, glycogenolysis, and gluconeogenesis. This review outlines current MR techniques to measure hepatic glucose metabolism. In addition to the methodological aspects of these techniques, (patho)physiological insights obtained by their applications in the healthy and diabetic liver during the past four decades are being presented.

## 2. MR Measurements of Liver Glucose and Glycogen Metabolism

### 2.1. ^13^C MRS

^13^C is a stable isotope of carbon with a natural abundance of 1.1%. In contrast to the most abundant isotope of carbon, i.e., ^12^C, ^13^C has a spin and can thus be detected with MR. However, the combination of low natural abundance and a gyromagnetic ratio (10.705 MHz/T) which is four times smaller than for ^1^H results in a low sensitivity for ^13^C, i.e., 0.0176% relative to ^1^H. Still, given adequate acquisition times, natural abundance ^13^C signals can be detected with MRS methods for liver metabolites present at high concentrations, such as lipids and glycogen. The advantage of the low signal of naturally abundant ^13^C spins is that ^13^C-enriched substrates, such as [1-^13^C]glucose, can be used to boost the signal and probe metabolic pathways. However, even with enrichment, ^13^C MRS at thermal polarization has a low intrinsic sensitivity, because of the aforementioned low gyromagnetic ratio. Moreover, as carbons in metabolites are mostly bound to hydrogens, signal intensity is further decreased by splittings caused by the ^13^C-^1^H scalar couplings when no ^1^H decoupling is applied. Therefore, to obtain a sufficient signal-to-noise ratio, ^13^C signals are usually acquired from a relatively large volume, using surface coil localization and/or slab selection with image-selected in vivo spectroscopy (ISIS) [6,7,8].

#### 2.1.1. Measurement of Hepatic Glycogen with ^13^C MRS

The detection of hepatic glucose is not feasible with ^13^C MRS at natural abundance, but the high concentration of glycogen in the liver can readily be detected even without enrichment, in spite of the high molecular weight of glycogen. This is because the high intramolecular mobility of the α-1,4-glycosidic linkage makes the C1 glycogen atom at 101 ppm completely MR visible. The [1-^13^C]glycogen signal has been used as a glycogen concentration marker in humans and animal models for a long time [9,10,11,12]. The T_1_ relaxation time of [1-^13^C]glycogen is strongly dependent on field strength, but relatively short T_1_ relaxation times (158 ± 15 ms at 4.7 T and 310 ± 10 ms at 8.4 T) [13], as compared to other ^13^C metabolites, allow for rapid signal averaging [14]. Both the T_1_ and T_2_ relaxation times of [1-^13^C]glycogen are not affected by the size of the glycogen chain, thus ensuring uniform visibility of ^13^C glycogen under varying physiological conditions [15]. ^1^H decoupling significantly increases the sensitivity of [1-^13^C]glycogen detection by collapsing the [1-^13^C]glycogen doublet signal into a singlet [14].

Although the within-session variability, i.e., repeatability, of hepatic glycogen content, as determined by ^13^C MRS, was reported to be low enough for monitoring physiological changes in hepatic glycogen levels [16], variability over longer periods, i.e., the reproducibility, also needs to be known for studies on the effects of longer-term interventions. To determine both the repeatability and reproducibility, glycogen concentrations were measured during 1 h after an overnight fast, during three visits within one month [16]. The repeatability of liver glycogen measurements within one session was significantly smaller than the reproducibility over 1 month (coefficients of variation (CV) of 13% vs. 35%), indicating variations in basal hepatic glycogen levels. However, later on, it was shown that a standardized preparation period, including a standardized diet and withdrawal from physical activity, could reduce the long-term (weeks) variability of hepatic glycogen content to the variability seen in a single session (long-term CV of 14.6% vs. single-session CV of 14.3%) [17].

**Figure 2:**
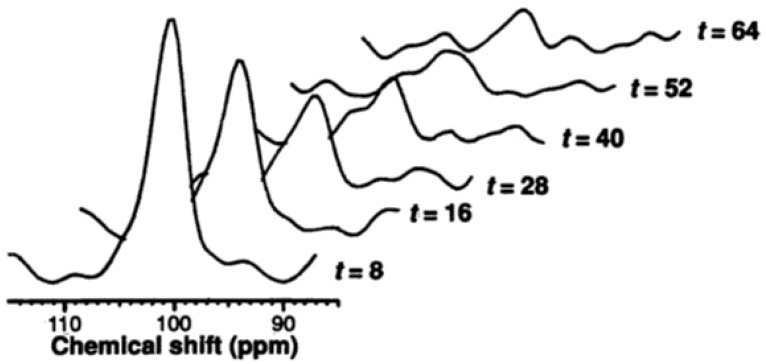
Longitudinal ^13^C MR measurements of hepatic glycogen at natural abundance in a healthy human subject during 64 h of fasting (at the indicated time points). ^1^H decoupling was applied during acquisition to remove the J-coupling-induced splitting of the [1-^13^C]glycogen resonance at 101 ppm. This figure was adapted from reference [7], with permission.

To understand the role of the liver in glucose homeostasis under different physiological conditions, hepatic glycogen concentrations have been monitored with ^13^C MRS upon interventions such as fasting, meal ingestion, and exercise. These experiments are commonly complemented with plasma glucose measurements for accurate modeling of glucose homeostasis. When these measurements include the analysis of plasma glucose enrichment after administration of, for example, deuterated or tritiated glucose, the rate of whole-body glucose production can be determined. Rothman et al. assessed the rate of net hepatic glycogenolysis in fasting healthy volunteers by measuring the [1-^13^C]glycogen signal in the liver during a 68 h fast, as exemplified in Figure 2 [7]. The net rate of gluconeogenesis was then calculated by subtracting the rate of net hepatic glycogenolysis from the rate of whole-body glucose production, as determined by plasma measurements. The mean rate of hepatic glycogenolysis was 4.3 ± 0.6 µmol/kg body weight per min during the first 22 h (corresponding to 36% of whole-body glucose production; throughout the remainder of the paper, the unit µmol/kg body weight per min is shortened to µmol/kg per min), but fell to 1.7 ± 0.5 and 0.3 ± 0.6 µmol/kg per min (18% and 4% of whole-body glucose production) during the 22–46 and 46–64 h intervals, respectively, while there were no significant changes in gluconeogenesis rates throughout the measurement (7.9 ± 1.0, 7.1 ± 0.5 and 8.3 ± 0.5 µmol/kg per min for 0–22, 22–46 and 46–64 h, respectively) [7]. In a subsequent study, the contributions of net hepatic glycogenolysis and gluconeogenesis to whole-body glucose production were determined in the earliest phase of the postabsorptive period, i.e., the first 12 h of a fast, when hepatic glycogen stores are at the maximum daily level. It was shown that, during the first 12 h of fasting, net hepatic glycogenolysis contributes about 45% to whole-body glucose production, meaning that more than 50% of glucose is produced by the gluconeogenesis [18].

During an overnight fast, liver glycogen stores were shown to be depleted by 32% [19], resulting in low glycogen concentrations in the morning (146–176 mM, as determined by nonlocalized ^13^C measurements [16,17,20], and 219–301 mM, as determined by using ISIS localization [8,21,22,23]). In studies focusing on the postprandial state, subjects are therefore usually fasted overnight, after which the repletion of liver glycogen is followed upon meal ingestion. Petersen et al. estimated the contribution of hepatic glycogen synthesis to whole-body glucose disposal after an oral glucose load upon an overnight fast and reported that 17% of the 98 g of ingested glucose was stored in the liver as glycogen over 180 min [24]. About 63% of the synthesized hepatic glycogen directly originated from glucose, while the rest was synthesized indirectly from three-carbon compounds [24]. As an alternative to a glucose load, mixed test meals that include carbohydrates, fats, and proteins have also been used in ^13^C MRS studies, as to mimic the normal response to daily meal intake [25]. It was shown that, after mixed meal ingestion, net hepatic glycogenesis continued up to 4 h, while net glycogenolysis was observed 6 h after the meal [25].

In addition to the assessment of hepatic glycogen replenishment after an overnight fast, exercise protocols have been employed to monitor post-exercise resynthesis of hepatic glycogen. While glycogen stores in skeletal muscle were shown to be significantly reduced after exercise (to exhaustion at 75% of maximal oxygen uptake) and partially restored after carbohydrate loading, no significant changes were observed in hepatic glycogen levels during exercise or subsequent carbohydrate loading in an early study [26]. This is in contrast with a later study in which the hepatic glycogen content was reduced by about 56 ± 2% after 83 min of cycling at 70% of maximal oxygen uptake [27]. Here, it was shown that the depleted hepatic glycogen stores could only be restored by the availability of exogenous carbohydrates, with the amount of resynthesis depending on the carbohydrate load [27].

#### 2.1.2. Measurement of Hepatic Glucose with ^13^C MRS

As it is not feasible to detect the natural abundance ^13^C signal of hepatic glucose in vivo, the administration of enriched [1-^13^C]glucose has been used to measure glucose in the liver [28]. When enriched [1-^13^C]glucose was administered by intravenous infusion under hyperglycemic–hyperinsulinemic clamp conditions, 10% of glucose was taken up by the liver, but this percentage increased to up to 30% with administration via the oral route [29]. The latter was explained by the first-pass effect, as after oral intake, glucose is taken up in the intestines and transported to the liver via the portal vein, before taking part in whole-body blood circulation. After being taken up into hepatocytes, glucose is converted to G6P by glucokinase. This phosphorylation could be detected by 0.14 and 0.16 ppm shifts in frequency of the C1 carbon resonances of β-glucose and α-glucose, respectively, in rat liver, allowing for the assessment of the hepatic G6P substrate cycle [30]. However, in human studies, these shifts are too small to be detected in vivo thus far. In the human liver, studies have monitored [1-^13^C]glucose and [1-^13^C]glycogen concentrations after enriched [1-^13^C]glucose administration to assess hepatic glycogenesis. After the oral administration of 75 g of glucose, of which 5 g was [1-^13^C]glucose, [1-^13^C]glucose and [1-^13^C]glycogen signals in the healthy liver reached peak concentrations at 60 and 204 min, respectively [31]. Recently, Stender et al. showed that state-of-the-art ^13^C RF coil arrays improved the signal-to-noise ratio (SNR) to an extent that MR spectroscopic imaging (MRSI) measurements of hepatic [1-^13^C]glycogen after oral administration of enriched [1-^13^C]glucose become feasible [32], thus offering insight into the spatial variations of glycogen storage within the liver.

### 2.2. Detecting Glucose and Glycogen in the Liver by Deuterium and Proton MRS/MRI

**Figure 3:**
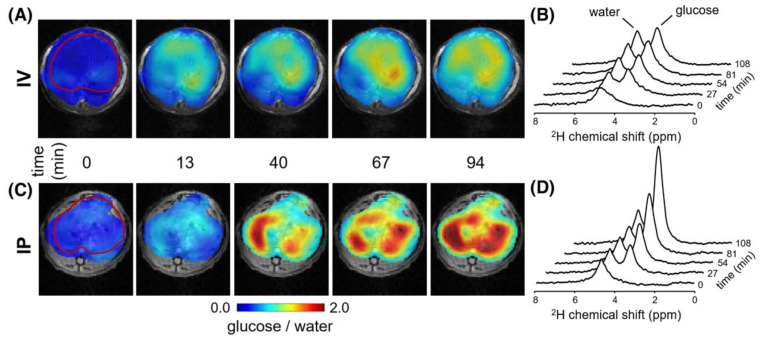
Metabolic maps of glucose/water ratio (**A**,**C**) and dynamic ^2^H spectra (**B**,**D**) in the rat liver (indicated by the red line in panels A and C for t = 0) after intravenous (IV) infusion (**A**,**B**) and intraperitoneal (IP) infusion (**C**,**D**) of [6,6′-^2^H_2_]glucose. Glucose signal reached higher intensities after IP infusion compared to IV infusion, as can be seen in both colormaps and dynamic ^2^H spectra. This figure was adapted from Reference [38], with permission.

As an alternative to ^13^C MRS with the administration of [1-^13^C]glucose, deuterium (^2^H)-labeled glucose has also been used to detect glucose uptake in the liver through ^2^H MRS. Deuterium is a stable isotope of hydrogen with a very low natural abundance (0.0115%) [33] and a gyromagnetic ratio of 6.5359 MHz/T. Because ^2^H nuclei are quadrupoles (spin 1), they have short T_1_ relaxation times [34,35], particularly compared to ^13^C nuclei [34,35], allowing fast signal averaging. This property has spurred the recent development of deuterium metabolic imaging (DMI) as a novel technique to study metabolism in vivo [36,37,38]. DMI relies on deuterium (^2^H) MRSI combined with the administration of deuterated compounds, such as deuterated glucose [36,37]. In the first DMI experiments conducted in the liver, the signal appearing at 3.8 ppm after administration of [6,6′-^2^H_2_]glucose was interpreted as a combination of deuterated glucose and deuterated glycogen, as they have overlapping resonances [36]. However, later it was shown that deuterated glycogen has such a short T_2_ relaxation time, that it is MR invisible in in vivo acquisitions, and that only deuterated glucose signal can be detected in the liver [38]. De Feyter et al. reported higher deuterated glucose signal intensity in the rat liver after intraperitoneal infusion compared to intravenous infusion (Figure 3), and this could be explained by the first-pass effect described above [38].

Chen et al. showed that glycogen H1 detection by ^1^H MRS with Nuclear Overhauser Enhancement (NOE) editing is a viable alternative to ^13^C MRS [39]. It is challenging to detect the H1 resonance of glycogen at 5.4 ppm in the liver, as it resonates close to the water frequency (4.7 ppm) and is overlapping with the intense olefinic lipid signal (5.4 ppm). NOE editing by inverting the glycogen H2 and H4′ resonances (3.6 ppm) enabled researchers to resolve the glycogen H1 peak from the residual water signal and overlapping lipid peak in a rat liver [39]. In the human liver, ^1^H MRS has also been applied for glycogen detection, but in this case, the H2H4′, H5, and H3 glycogen signals at 3.65, 3.81, and 3.94 ppm, respectively, were measured and quantified, using the liver water signal as an internal reference [40]. Respiratory gating was performed to suppress motion-related artifacts [40]. The liver glycogen content could be quantified in 25 out of 46 healthy volunteers. Although the ^1^H MRS measurements were performed 2–3 h after meal intake, glycogen concentrations were much lower, i.e., 38.1 ± 14.4 mmol/kg wet weight (based on a specific gravity of the liver of 1.054 kg/L [41], corresponding to 40.2 ± 15.2 mM), compared to reported ^13^C MRS results 4 h after meal intake (350 ± 18 mM) [25,40]. The apparent underestimation of glycogen concentration by ^1^H MRS may at least partially be explained by an overestimation of T_2_. Weis et al. [42] reported much shorter T_2_ values for glycogen H2/H4′, H5, and H3 compared to the previous study [40], i.e., 13 ± 4 ms vs. 36 ± 8 ms, and measured hepatic glycogen concentrations of 145 ± 50 mM a few hours after lunch, using ^1^H MRS [42]. Rather than quantifying glycogen from ^1^H MR spectra, van Zijl et al. demonstrated that chemical exchange saturation transfer (CEST) techniques can be used to image glycogen in the liver with ^1^H MRI [43]. In such a glycoCEST experiment, protons of the hydroxyl groups in the glycogen chain are saturated with RF pulses, and, as the saturated protons exchange with water protons, partial saturation is observed for the water signal. However, the application of glycoCEST MRI in the in vivo liver has proven to be challenging because of the fast exchange of glycogen hydroxyl protons and the overlap with other hydroxyl moieties. Recently, it was shown that the NOE signal on the other side of the water resonance in the Z-spectrum, attributed to the glycogen H2/H4′, H5, and H3 resonances, may be more viable for in vivo liver glycogen imaging [44].

## 3. ^13^C MRS Investigations of Impairments of Hepatic Glucose and Glycogen Metabolism in Diabetes

The noninvasive nature of MRI/MRS methods makes them suitable tools to apply in studies of metabolic diseases without much burden for the patient. The methods mentioned in the previous section have been used to understand the impairment of hepatic glucose and glycogen metabolism in diabetes.

### 3.1. Type 2 Diabetes

In one of the early studies, Magnusson et al. utilized ^13^C MRS to measure hepatic glycogenolysis and gluconeogenesis in fasting T2D patients and healthy controls from 4 to 23 h after meal ingestion [45]. At 4 h after meal ingestion, the hepatic glycogen concentration was lower in the diabetic group compared to the control group (131 ± 20 vs. 282 ± 60 mM), and over the 23 h of fasting, net hepatic glycogenolysis was significantly decreased in the T2D patients compared to controls (1.3 ± 0.2 vs. 2.8 ± 0.7 µmol/kg per min) [45]. Whole-body glucose production was also measured as described before and was found to be higher in the T2D patients than in healthy controls. From the difference between whole-body glucose production and net hepatic glycogenolysis, it was determined that gluconeogenesis was consequently also higher in the T2D patients compared to the controls (9.8 ± 0.7 vs. 6.1 ± 0.5 µmol/kg per min) and that increased gluconeogenesis therefore accounts for the increased whole-body glucose production in T2D patients during fasting [45]. Tomiyasu et al. measured hepatic glycogen levels after oral administration of 75 g of glucose, of which 5 g was [1-^13^C]glucose, and observed that, in contrast to healthy controls, in most T2D patients, there was no clear increase in the hepatic glycogen signal, indicating negligible glycogenesis in the postprandial state [31]. In a comprehensive study, Krššák et al. investigated hepatic glycogen metabolism and endogenous glucose production (EGP) in T2D patients in preprandial and postprandial conditions (before and after dinner) [46]. Before dinner, hepatic glycogen concentrations measured with ^13^C MRS were lower in T2D patients compared to the healthy controls (227 ± 6 vs. 275 ± 10 mM), and after meal ingestion, net rates of hepatic glycogenesis were significantly reduced in T2D patients compared to the controls (0.76 ± 0.16 vs. 1.36 ± 0.15 mg/kg per min) [46]. Moreover, both before and after dinner, EGP was higher in the T2D group compared to controls, and maximum suppression of EGP occurred much later in T2D patients, demonstrating that both decreased net hepatic glycogenesis and delayed of suppression of EGP contribute to postprandial hyperglycemia in T2D patients [46]. Importantly, these impairments were not normalized under conditions of hyperinsulinemic hyperglycemia, showing that insulin resistance plays an important role in the defective hepatic glycogen metabolism in T2D [46].

Mutations in the glucokinase gene lead to a subtype of diabetes, termed maturity onset diabetes of young 2 (MODY 2). Using ^13^C MRS, it was shown that, while fasting hepatic glycogen concentrations were similar between MODY-2 patients and controls, glucokinase-deficient MODY-2 patients had a 30–60% lower net increment of hepatic glycogen and relatively elevated hepatic gluconeogenesis after meals [47]. The former likely results from impaired glucose phosphorylation in hepatocytes due to the glucokinase deficiency, and both glycogenesis and gluconeogenesis abnormalities may contribute to the hyperglycemia in these patients.

The first-line therapy for T2D is treatment with metformin, which is an oral medication which reduces glucose production by the liver [48]. Hundal et al. examined T2D subjects before and after 3 months of metformin treatment to assess whether metformin reduces hepatic glucose production by reducing gluconeogenesis or by reducing hepatic glycogenolysis [49]. Before metformin treatment, EGP was 2-fold higher, the rate of net hepatic glycogenolysis determined by ^13^C MRS was 40% lower, and the derived rate of gluconeogenesis was more than 3-fold higher in T2D patients compared to controls. Metformin treatment of T2D patients reduced EPG by 25% but tended to increase the rate of net hepatic glycogenolysis. Therefore, metformin lowered EGP in T2D patients through a reduction in gluconeogenesis (37% decrease in the rate of gluconeogenesis) [49]. Since recently, sodium-glucose cotransporter-2 (SGLT-2) inhibitors are increasingly being considered as a first-line oral treatment for T2D, especially in patients with (an increased risk of) cardiovascular disease [50]. SGLT-2 inhibitors improve whole-body glucose homeostasis, although their use is also associated with a significant rise in EGP [51]. The contribution of hepatic glycogenolysis to increased EGP with the SGLT-2 inhibitor dapagliflozin was studied in healthy volunteers and T2D patients with ^13^C MRS [52]. Subjects were investigated twice, once with a placebo and once with dapagliflozin (2 weeks apart), and scans were made before and (90–180 min and 300–390 min) after administration of placebo/dapagliflozin after an overnight fast. Rates of net hepatic glycogenolysis were significantly lower in T2D patients compared to controls but were not affected by dapagliflozin in either group. EGP was only increased in healthy controls after dapagliflozin administration and not in T2D patients, and the same was observed for the derived rates of gluconeogenesis. From this study, it was concluded that the rise in EGP after SGLT-2 inhibition in healthy controls is due to increased gluconeogenesis, either in the liver or in the kidneys, but not due to an increase in glycogenolysis [52].

### 3.2. Type 1 Diabetes

In addition, in T1D, the impaired glycemic response in the postprandial state has been linked with both defective hepatic glycogen synthesis and elevated rates of gluconeogenesis. ^13^C MRS has been used to monitor hepatic glycogen concentrations in poorly controlled T1D patients throughout the day, starting from before breakfast and continuing up to 4 h after dinner [8]. In contrast to T2D patients, hepatic glycogen concentrations after an overnight fast were not significantly lower in T1D patients compared to controls, i.e., 250 ± 30 vs. 274 ± 11 mM [8]. However, whereas hepatic glycogen increased by 144 ± 14 mM in controls throughout the day, it only increased by 44 ± 20 mM in T1D patients, showing a major defect in net hepatic glycogen synthesis [8]. In addition, hepatic gluconeogenesis was augmented in the poorly controlled T1D patients [8]. T1D is one of the most common metabolic diseases seen in children [53]. Liver glycogen content has been measured in children with T1D (on conventional insulin regimens) and matched controls both in the fasting (morning) and fed (afternoon) state, using ^13^C MRS [54]. Children with T1D tended to have lower fasting glycogen values than the controls, but liver glycogen increased in all children with T1D during the day and reached similar values as in the controls in the afternoon. Thus, in contrast to adults with T1D, glycogen synthesis was not impaired in children with T1D [54].

Bischof et al. showed that the defective hepatic glycogen metabolism in adult T1D patients can be improved by intense insulin treatment [21,22]. While ^13^C MRS-measured rates of net glycogen synthesis and net glycogen breakdown were reduced (∼74% and ∼47%, respectively) in poorly controlled T1D patients compared to controls, short-term (24 h) intense insulin treatment increased both glycogen synthesis and glycogen breakdown rates, although not to the level of healthy controls [21]. Long-term intensive insulin therapy (with multiple daily insulin injections, using the basal-bolus principle) even completely normalized glycogen synthesis and breakdown, as well as the rate of gluconeogenesis, in T1D patients [22]. Insulin-pump therapy, also known as continuous subcutaneous insulin infusion, imitates the physiological delivery of insulin by employing a portable pump to continuously administer insulin at a slow, basal rate for 24 h, with patient-activated boosts when food is consumed. It was shown that insulin-pump therapy, like long-term intensive insulin therapy with injections, also completely restores net glycogen synthesis and gluconeogenesis rates in T1D patients [20]. Castle et al. studied how T1D patients receiving insulin-pump therapy react to repeated small doses of glucagon [55]. Both in the fasted and in the fed state, ^13^C MRS-measured hepatic glycogen levels were not significantly affected by glucagon, supporting the safety of repeated small-dose glucagon delivery in a bihormonal closed-loop system [55].

### 3.3. Glycogen Storage Disease

Abnormalities in hepatic glycogen metabolism are seen not only in diabetes, but also in other metabolic diseases, such as glycogen storage disease. ^13^C MRS has been applied in a patient with type Ia glycogen storage disease, showing a two-fold higher hepatic glycogen concentration compared to healthy controls [56].

## 4. Probing Gluconeogenesis and Glycolysis with Hyperpolarized ^13^C MR

### 4.1. Hyperpolarized ^13^C MRS

Up to this point, MRI and MRS techniques have been covered that utilize the MR signal at thermal equilibrium polarization. As the sensitivity of these techniques is rather low, it is not feasible to detect metabolite pools at low concentrations. Hyperpolarization of ^13^C-labeled substrates with dynamic nuclear polarization (DNP) increases the SNR by a factor of 10,000 or more by increasing the available net magnetization for the ^13^C MR experiment, although only for a limited time of signal acquisition [57]. DNP involves the mixing of the ^13^C-labeled substrate with a radical, of which the free electrons become nearly 100% polarized when placed in a magnetic field at a very low temperature (1 K). The polarization of the electron spins is then transferred to the ^13^C nuclear spins through microwave irradiation. After rapid dissolution of the sample, it can be injected intravenously. The development of DNP enabled real-time molecular and metabolic imaging [57,58,59]. However, because the increased sensitivity is relatively short-lived (typical T_1_ relaxation times are 60 s or less), hyperpolarized ^13^C MR is limited to the detection of rapid metabolism.

### 4.2. Use of Hyperpolarized [2-^13^C]Dihydroxyacetone to Simultaneously Assess Gluconeogenesis and Glycolysis

The direct detection of glucose as a product of gluconeogenesis with MRS remains a challenge. In the studies cited above, rates of gluconeogenesis were calculated indirectly from the difference between the rate of EGP, determined from plasma measurements, using tracer dilution methods, and the rate of hepatic glycogenolysis, measured by ^13^C MRS. The use of hyperpolarized [2-^13^C]dihydroxyacetone (DHA) offers a new approach to probe gluconeogenesis directly in the liver in real time [60]. DHA is rapidly phosphorylated to dihydroxyacetone phosphate (DHAP), an intermediate 3-carbon molecule in both gluconeogenesis and glycolysis. After injection of [2-^13^C]DHA in the perfused mouse liver, products of both gluconeogenesis (hexoses) and glycolysis (3-carbon intermediates) became rapidly enriched [60]. The ratio of ^13^C-labeled 3-carbon intermediates to hexoses appeared to be a sensitive (inverse) index of gluconeogenesis, showing a ~5-fold decrease between glycogenolytic and gluconeogenic conditions [60]. The sensitivity of hyperpolarized [2-^13^C]DHA to monitor hepatic gluconeogenic flux was further demonstrated in a study conducted on perfused livers from healthy mice treated with either glucagon or metformin and perfused livers from diabetic mice [61]. The ratio of ^13^C-labeled hexoses to 3-carbon intermediates (note that an inverse relation was used compared to the previous study) was more than three times larger in the diabetic and glucagon-treated livers compared to the metformin-treated livers [61], which is in agreement with the expected elevated gluconeogenesis in the diabetic liver and the promotion of gluconeogenesis by glucagon, respectively. Although initial results were promising, technical challenges, such as the requirement of large bandwidth RF pulses and ^1^H decoupling, limited in vivo applications of hyperpolarized [2-^13^C]DHA. However, with improvements in the RF pulse design, the feasibility of using hyperpolarized [2-^13^C]DHA to assess gluconeogenesis and glycolysis in vivo was recently also demonstrated in mice [62].

### 4.3. Use of Hyperpolarized [1-^13^C]Pyruvate to Assess Gluconeogenesis and PDH Flux

**Figure 4:**
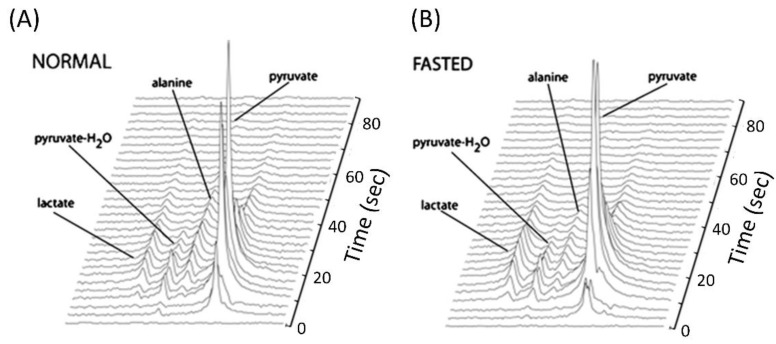
Dynamic hyperpolarized ^13^C MR spectra of the in vivo liver of fed (**A**) and fasted rats (**B**). Acquisition started after intravenous administration of hyperpolarized [1-^13^C]pyruvate, and spectra were collected every 3 s. The production of [1-^13^C]alanine was significantly lower in the fasted liver compared to that in the fed state. This figure was adapted from Reference [63], with permission.

Hyperpolarized [1-^13^C]pyruvate is a popular substrate for metabolic imaging studies because it is at the crossroads of different metabolic pathways (Figure 1). Moreover, the relatively long T_1_ relaxation time of hyperpolarized [1-^13^C]pyruvate allows for a longer acquisition window compared to other hyperpolarized compounds. Hu et al. demonstrated the feasibility of using [1-^13^C]pyruvate to assess liver metabolism in rats in vivo and to detect metabolic differences between fasted and fed conditions [63]. Upon injection of hyperpolarized [1-^13^C]pyruvate, rapid formation of [1-^13^C]lactate and [1-^13^C]alanine was observed (Figure 4), but the production of [1-^13^C]alanine was significantly lower in the fasted liver compared to that in the fed state [63], and this could be interpreted to reflect the gluconeogenic state of the fasted liver. The reduced labeling of [1-^13^C]alanine (and of [1-^13^C]lactate) was also observed in the in vivo livers of fasted mice compared with hyperinsulinemic–isoglycemic clamped mice [64] and in perfused livers of fasted mice compared with fed mice [5]. In the latter study, in addition to [1-^13^C]lactate and [1-^13^C]alanine, also the production of hyperpolarized [1-^13^C]malate, [4-^13^C]malate, [1-^13^C]aspartate, [4-^13^C]aspartate, and [1-^13^C]bicarbonate from hyperpolarized [1-^13^C]pyruvate was detected [5]. The labeling of malate and aspartate signifies the rapid carboxylation of pyruvate to oxaloacetate by PC, the first step in the conversion of pyruvate to glucose. The appearance of these intermediates was surprisingly not different between fed and fasted states, but it was considerably blunted in PEPCK knockout mice, which cannot synthesize glucose from pyruvate and have a severely impaired PC flux [5]. Hyperpolarized [1-^13^C]bicarbonate can be generated from hyperpolarized [1-^13^C]pyruvate by flux through both PDH and PC; however, an isotopomer analysis revealed that PC flux was more than 7-fold greater than PDH flux in the livers of wild-type mice. In accordance, [1-^13^C]bicarbonate was not detected in PEPCK knockout mice [5]. The dominance of PC flux over PDH flux in the liver was also demonstrated with thermally polarized ^13^C NMR, using standard isotopomer methods [65]. In contrast, using a combination of in vivo experiments with hyperpolarized [1-^13^C]pyruvate and ex vivo experiments with non-hyperpolarized [2,3-^13^C]pyruvate, Jin et al. showed that, whereas PC flux is indeed dominant under fasting conditions, PDH flux is dominant in fed conditions and that the production of [1-^13^C]bicarbonate from hyperpolarized [1-^13^C]pyruvate in the liver of fed rats exclusively reflects PDH flux [66]. In accordance, stimulation of PDH by insulin during an hyperinsulinemic–isoglycemic clamp increased the labeling of [1-^13^C]bicarbonate in mouse livers [64].

Hyperpolarized [1-^13^C]pyruvate has also been used to measure gluconeogenesis and PDH flux in the liver of animal models of diabetes. In a high-fat-diet-induced mouse model of T2D, the production of hyperpolarized [1-^13^C]malate and [1-^13^C]aspartate from hyperpolarized [1-^13^C]pyruvate was higher compared to that of the control mice, signifying an increased PC flux, an important pathway for increased gluconeogenesis in diabetes [67]. Metformin treatment of the high-fat-diet-fed mice successfully reduced hepatic gluconeogenesis, as evidenced by lower malate and aspartate labeling [67]. Because diabetes is a systemic disease that affects multiple tissues in the body, simultaneous measurement of metabolism in different organs is relevant for understanding the disease mechanism. For example, simultaneous measurement of pyruvate metabolism in the liver and heart can inform about the interplay between these two organs in diabetes, especially in the context of cardiovascular disease [68]. To this end, the feasibility of interleaved ^13^C MRS measurements localized to the liver and heart, respectively, upon injection of hyperpolarized [1-^13^C]pyruvate has been shown in a rat model of T2D [69]. PDH fluxes were shown to be decreased in diabetic rats compared with control rats, in both the heart and the liver [69]. In addition, the production of [1-^13^C]alanine was reduced in the liver of diabetic rats, but not in the heart, supporting the gluconeogenic state of the diabetic liver [69]. Gluconeogenesis not only takes place in the liver, but also (to a lesser extent) in the kidneys, and renal glucose release is significantly elevated in diabetes [70]. Von Morze et al. demonstrated the application of localized hyperpolarized ^13^C MRS simultaneously in the liver and kidneys of rats [71,72]. With hyperpolarized [1-^13^C]pyruvate, the production of hyperpolarized [1-^13^C]bicarbonate and [1-^13^C]alanine was lower in both the liver and kidneys of fasted rats compared to control rats [71]. A similar observation was made in a rat model of T1D, supporting the increased gluconeogenesis in both diabetic liver and kidneys [71]. In contrast, in T2D rats, the production of hyperpolarized [1-^13^C]alanine from hyperpolarized [1-^13^C]pyruvate in the liver and kidneys was elevated compared to controls, while, in both tissues, [1-^13^C]lactate was similarly increased in T1D and T2D rats [72]. Thus, hyperpolarized ^13^C MRS can be a powerful tool to noninvasively characterize phenotypic differences simultaneously in multiple tissues in vivo.

## 5. Limitations and Future Perspective

Apart from the inherent methodological limitations already covered above, there are some challenges that are worth mentioning. MR is an insensitive technique, and labeled compounds such as [1-^13^C]glucose are very costly at the amount required for human studies. In comparison to [1-^13^C]glucose, [6,6′-^2^H_2_]glucose is a cheaper alternative, yet the detection of glycogen labeling is not possible with [6,6′-^2^H_2_]glucose [38].

The studies using hyperpolarized ^13^C-labeled substrates discussed in this review were all performed in small animal models. Although, hyperpolarization of ^13^C-labeled substrates gives a unique opportunity to detect low concentration metabolites, due to the high costs of clinical polarizers and of Good Manufacturing Practice doses of hyperpolarized substrates and the challenges related to the robustness, reliability, and efficiency of hyperpolarized ^13^C MRS experiments, applications in humans have remained limited so far [73]. In addition, hyperpolarized ^13^C MRS in the human liver is very challenging due to the limited coverage with commonly used ^13^C surface coils, physiologic motion (in particular respiratory motion), and B_0_ inhomogeneities over a large field of view. These limitations were overcome by recent developments, and the first results showed the feasibility of whole-abdomen (liver, pancreas, spleen, and kidneys) hyperpolarized [1-^13^C]pyruvate metabolic imaging [74].

The development of ultra-high field (UHF) scanners (≥ 7 T) has increased the sensitivity of liver MRS, as the SNR is proportional to the main magnetic field strength [75,76]. Consequently, the number of required averages, or acquisition time, to achieve an adequate SNR is lower at UHF. In preliminary work, Poli et al. showed the feasibility of interleaved acquisitions of DMI and ^13^C MRS at UHF to monitor both hepatic glucose uptake and glycogen accumulation in real time [77]. Recently, it was shown that, in addition to monitoring hepatic glucose uptake, DMI has the potential to also simultaneously measure gastric emptying and renal glucose metabolism with the use of a dedicated setup at UHF [78,79]. Such combined measurements can give insight into the interplay between disturbances in postprandial liver and kidney glucose metabolism and gastric emptying in patients with diabetes.

## 6. Conclusions

For four decades, MRI and MRS methods have allowed for the assessment of glucose metabolism in the liver in a noninvasive manner. Table 1 summarizes the techniques covered in this review and the insights obtained by their application in both the healthy and diabetic liver. MR methods based on the detection of ^13^C, ^1^H, or ^2^H nuclei at thermal polarization, either at natural abundance (for ^13^C and ^1^H) or upon the administration of isotopically enriched substrates (for ^13^C and ^2^H), enabled the dynamic or longitudinal measurement of hepatic glycogen and glucose concentrations under a variety of physiological conditions, such as during fasting, after meal intake, or after exercise, providing insight into rates of hepatic glycogenesis, glycogenolysis, and (indirectly) gluconeogenesis. The development of hyperpolarized ^13^C MR paved the way to detect intermediates of liver glucose metabolism present at lower concentrations, although only for a short window of acquisition time, enabling the direct assessment of hepatic glycolysis and gluconeogenesis. Understanding the underlying mechanisms of impaired glucose metabolism in the liver becomes ever more important as the prevalence of diabetes increases. Technical improvements of the MR methods not only extend our insight into (distortions of) hepatic metabolic pathways, but also lead the transition from fundamental research to the personalized treatment of patients.

## Figures and Tables

**Table 1:** MR techniques for assessing hepatic glucose metabolism in vivo and obtained insights in both the healthy and diabetic liver.

Technique	Measurement	Metabolic Process in the Liver	Insights	References
^13^C MRS	Liver glycogen levels after meal/glucose intake	Glycogenesis	In healthy subjects, about 17% of an oral glucose load is stored as glycogen in the liver and net hepatic glycogenesis continues up to 4 h after meal intake.	[24,25]
			Hepatic glycogenesis is reduced in T2D, MODY, and (adult) T1D patients.	[8,21,31,45,46,47]
			(Long-term intensive) insulin treatment restores (or even normalizes) glycogenesis in T1D patients.	[20,21,22]
	Liver glycogen levels during fasting	Glycogenolysis	In healthy subjects, the contribution of hepatic glycogenolysis to whole-body glucose production is ~45% during the first period of fasting, and it gradually declines thereafter.	[7,18]
			Hepatic glycogenolysis is reduced in T2D and T1D patients.	[21,45,46,49,52]
			Metformin tends to increase glycogenolysis in T2D patients, but the SGLT-2 inhibitor dapagliflozin has no effect on glycogenolysis.	[49,52]
			(Long-term intensive) insulin treatment restores (or even normalizes) glycogenolysis in T1D patients.	[21,22]
	Liver glycogen levels during fasting, in combination with plasma measurements of whole body glucose production	Gluconeogenesis	In healthy subjects, the contribution of hepatic gluconeogenesis to whole-body glucose production during early fasting is more than 50% and remains relatively constant with longer fasting.	[7,18]
			Hepatic gluconeogenesis is elevated in T2D, MODY, and T1D patients.	[8,45,46,47,49,52]
			Metformin reduces gluconeogenesis in T2D patients, but the SGLT-2 inhibitor dapagliflozin has no effect on gluconeogenesis in T2D patients, while it increases gluconeogenesis in healthy subjects.	[49,52]
			Long-term intensive insulin treatment normalizes gluconeogenesis in T1D patients.	[20,22]
^13^C MRS/DMI	Liver glucose levels after [1-^13^C]glucose/[6,6′-^2^H_2_]glucose administration, either orally/IP or IV	Glucose uptake	Hepatic glucose uptake is higher after oral/IP glucose administration compared to IV infusion.	[29,38]
Hyperpolarized ^13^C MRS	^13^C labeling of hexoses relative to 3-carbon intermediates after infusion of [2-^13^C]DHA	Gluconeogenesis (vs. glycolysis)	Hepatic gluconeogenesis is elevated under fasting conditions and in a T2D animal model.	[60,61]
	^13^C labeling of [1-^13^C]alanine and [1-^13^C]lactate after infusion of [1-^13^C]pyruvate	Gluconeogenesis	Hepatic gluconeogenesis is elevated under fasting conditions and in T2D and T1D animal models.	[5,63,64,69,71,72]
	^13^C labeling of [1-^13^C]malate and [1-^13^C]aspartate after infusion of [1-^13^C]pyruvate	PC flux (gluconeogenesis)	Hepatic PC flux is elevated in a T2D animal model and can be reduced with metformin.	[67]
	^13^C labeling of [1-^13^C]bicarbonate after infusion of [1-^13^C]pyruvate	PC and/or PDH flux	Under fasting conditions, PC flux is dominant in the liver, but PDH flux is dominant in fed conditions.	[5,64,66]
			Hepatic PDH flux is decreased in a T2D animal model.	[69]

DMI, deuterium metabolic imaging; DHA, dihydroxyacetone; IP, intraperitoneal; IV, intravenous; PC, pyruvate carboxylase; PDH, pyruvate dehydrogenase; T1D, type 1 diabetes; T2D, type 2 diabetes.
